# Childhood diarrheal morbidity and sanitation predictors in a nomadic community

**DOI:** 10.1186/s13052-017-0412-6

**Published:** 2017-10-06

**Authors:** Bikes Destaw Bitew, Wondwoson Woldu, Zemichael Gizaw

**Affiliations:** 1 Department of Environmental and Occupational Health and Safety, Institute of Public Health, College of Medicine and Health Sciences, University of Gondar, Gondar, Ethiopia; 2 Hadaleala District Health Office, Hadaleala District, Hadaleala town, Afar Regional State Ethiopia

**Keywords:** Childhood diarrheal disease, Under-five children, Environmental sanitation, Nomadic people, Multi variable binary logistic regression, Hadaleala district, Ethiopia

## Abstract

**Background:**

Diarrhea remains a leading killer of young children on the globe despite the availability of simple and effective solutions to prevent and control it. The disease is more prevalent among under – five children (U5C) in the developing world due to lack of sanitation. A child dies every 15 s from diarrheal disease caused largely by poor sanitation. Nearly 90% of diarrheal disease is attributed to inadequate sanitation. Even though, the health burden of diarrheal disease is widely recognized at global level, its prevalence and sanitation predictors among a nomadic population of Ethiopia are not researched. This study was therefore designed to assess the prevalence of childhood diarrheal disease and sanitation predictors among a nomadic people in Hadaleala district, Afar region, Northeast Ethiopia.

**Methods:**

A community based cross-sectional study design was carried out to investigate diarrheal disease among U5C. A total of 704 households who had U5C were included in this study and the study subjects were recruited by a multistage cluster sampling technique. Data were collected using a structured questionnaire and an observational checklist. All the mothers of U5C found in the selected clusters were interviewed. Furthermore, the living environment was observed. Univariable binary logistic regression analysis was used to choose variables for the multivariable binary logistic regression analysis on the basis of p- value less than 0.2. Finally, multivariable binary logistic regression analysis was used to identify variables associated with childhood diarrhea disease on the basis of adjusted odds ratio (AOR) with 95% confidence interval (CI) and *p* < 0.05.

**Results:**

The two weeks period prevalence of diarrheal disease among U5C in Hadaleala district was 26.1% (95% CI: 22.9 - 29.3%). Childhood diarrheal disease was statistically associated with unprotected drinking water sources [AOR = 2.449, 95% CI = (1.264, 4.744)], inadequate drinking water service level [AOR = 1.535, 95% CI = (1.004, 2.346)], drinking water sources not protected from animal contact [AOR = 4.403, 95% CI = (2.424, 7.999)], un-availability of any type of latrine [AOR = 2.278, 95% CI = (1.045, 4.965)], presence of human excreta in the compound [AOR = 11.391, 95% CI = (2.100, 61.787)], not washing hand after visiting toilet [AOR = 16.511, 95% CI = (3.304, 82.509)], and live in one living room [AOR = 5.827, 95% CI = (3.208, 10.581)].

**Conclusion:**

Childhood diarrheal disease was the common public health problem in Hadaleala district. Compared with the national and regional prevalence of childhood diarrhea, higher prevalence of diarrhea among U5C was reported. Types of drinking water sources, households whose water sources are shared with livestock, volume of daily water collected, availability of latrine, presence of faeces in the compound, hand washing after visiting the toilet and number of rooms were the sanitation predictors associated with childhood diarrhea. Therefore, enabling the community with safe and continuous supply of water and proper disposal of wastes including excreta is necessary with particular emphasis to the rural nomadic communities.

## Background

Simple and effective solutions are available to prevent and control diarrheal disease.However, the disease remains a leading killer of young children on the globe, accounting for 9% of all deaths among U5C worldwide in 2015. This translates into over 1400 young children dying each day, or about 530,000 children a year. Most child deaths from diarrhea occur in South Asia and sub-Saharan Africa [1–3].

Diarrhea is more prevalent in the developing world due to the lack of safe drinking water, sanitation and hygiene, as well as poorer overall health and nutritional status. According to the latest available figures, an estimated 2.4 billion people lack improved sanitation facilities, and nearly 1 billion people do not have access to safe drinking water. These unsanitary environments allow diarrhea causing pathogens to spread more easily [4].

Diarrheal disease is a serious public health problem among U5C in developing countries. The rate of diarrheal infections in many developing countries is extremely high. Diarrheal pathogens are constantly being excreted into the environment and in massive quantities. As a direct consequence, in Ethiopia about 13% of children under age of 5 years had diarrhea [5]. In the country, 24 - 30% of all infant deaths and 25% of deaths among children aged between 1 and 4 years were due to diarrhea [6]. Similarly, diarrheal disease was also the major cause of 12.7% under-five mortality in the Afar region, where the area of the current study is located [5].

Studies all over the world illustrated that childhood diarrhea is the result of many factors. However, the overwhelming causes are associated with poor sanitation. Diarrheal disease due to lack of sanitation is the greatest cause of morbidity and mortality among U5C in the world, especially in poor countries [7–10]. A child dies every 15 s from diarrheal disease caused largely by poor sanitation [11]. Nearly 90% of diarrheal disease is attributed to inadequate sanitation [8, 12].

Although, the health burden of diarrheal disease is widely recognized at global level, its prevalence and sanitation predictors among the nomadic populations of Ethiopia are not researched. This study was therefore designed to assess the prevalence of childhood diarrheal disease and sanitation predictors among nomadic people in Hadaleala district, Afar region, Northeast Ethiopia.

## Methods

### Study design and description of study settings

A community based cross-sectional study was conducted in Hadaleala district, Afar region, Northeast Etiopia from April 28 to May 17, 2015. Hadaleala district is one of the districts found in Hariresu zone, the Afar National Regional State. It is located at 341 km to the Southwest direction from the regional capital city Semera and 268 km away to the North of Addis Ababa, the capital of Ethiopia. In the district, an estimated 7516 households were found with an average household size of 5.7 persons per house and with an U5C population of 10.1%. The population lives in a very scattered manner where the average population density is 14 persons/ km^2^, which makes the establishment of basic sanitation facilities difficult. The community who lives in all kebeles (the lowest administrative units) of the district is mobile pastoralist. The community suffers from a shortage of water, hygiene and sanitation facilities. During 2015, safe water and sanitation coverage of the district was 35% and 12%, respectively [13].

### Sample size determination

The sample size was determined using single population proportion formula by considering these assumptions: *p* = 31% (the two weeks period prevalence of diarrhea among U5C in Arbaminch district) [14], 95% confidence interval, 5% margin of error (d),

$$ n=\frac{{\left({z}_{\raisebox{1ex}{$\alpha $}\!\left/ \!\raisebox{-1ex}{$2$}\right.}\right)}^2p\left(1-p\right)}{d^2}=\frac{(1.96)^20.31\left(1-0.31\right)}{0.05^2}=328 $$

Considering the design effect of 2 and 5% non response rate, the final sample size was 689 mothers-child pair.

### Sampling procedures

For the reason that the study participants are nomadic and scattered, multistage cluster sampling technique was used to select study participants. The clusters were villages with defined geographical boundaries. From a total of 11 kebeles, 6 were randomly selected by a lottery method. The selected 6 kebeles were clustered into 39 villages and from these villages, 17 were selected by systematic random sampling technique. Finally, all (704) households who had U5C were included in the study. For households having more than one child, the younger one was taken as index child and recruited in the study.

### Measurement of study variables

Childhood diarrheal disease, which was the outcome variable of this study was defined as having three or more loose or watery stools within 24 h period [15, 16]. The prevalence of diarrhea within the two weeks period prior to data collection was calculated as the total number of diarrhea cases divided by the total number of U5C in the study area. The predictor variables were defined based on the minimum or basic indicators for access to basic sanitation set by the World Health Organization (WHO) and United Nations Children’s Fund (UNICEF) [17].

### Data collection tools and procedures

A structured questionnaire and an observational checklist were used to collect data. The tools were prepared in English language and translated to the local language and back translated to English to maintain the consistency of the questions. Prior to the actual data collection period, the tools were pre-tested out of the study area. To increase quality of data, eight diploma nurses and two Environmental Health Officers who were fluent in speaking both Amharic and Afarigna language and working in the district were involved in the data collection process. After pretest and training of data collectors, the collectors had visited all households found in the selected clusters. When collectors got U5C during the visit, they asked the mothers and observed the living environment. The younger children were included in the study when there were more than one U5C in the household. Finally, the collected data were checked and corrected by the data collectors immediately after finalizing the questionnaire. Supervisors had also checked the completeness of all the daily filled questionnaires and 5% of the collected questionnaires were repeated.

### Data management and statistical analysis

Data were entered using EPI-INFO version 3.5.3 statistical package and exported into SPSS version 20.0 for further analysis. For most variables, data were presented by frequencies and percentages. Univariable binary logistic regression analysis was used to choose variables for the multivariable binary logistic regression analysis and variables which had a p – value less than 0.2 by the univariable analysis were then analyzed by multivariable binary logistic regression for controlling the possible effect of confounders and finally, variables which had association were identified on the basis of AOR with 95% CI and a *p* < 0.05.

## Results

### Sanitation condition in the nomadic community

Drinking water sources and water handling at home, excreta management, solid waste disposal, and housing conditions were assessed by the current study. The majority, 453 (64.3%) of households had collected water from unprotected water sources. Three hundred seventy-three (53.0%) households had collected water below 15 L/C/d. More than two - third, 488 (69.3%) of the households stored drinking water in wide mouthed containers and almost all of the households did not properly cover the storage containers at the time of the survey. Six hundred eighty-four (97.2%) of the households drew water from the storage container by pouring. A small proportion (4.8%) of the households practiced home based water treatment. More than three fold, 563 (80.0%) of the households didn’t have latrines. Residents of six hundred thirty-seven (90.5%) households did not wash their hands after visiting toilet. Moreover, the great majority of the households, 621 (88.2%) had practiced indiscriminate waste disposal. Five hundred ninety-four (84.4%) of the households lived in a house with earthen floor and most, 398 (56.5%) of the dwellings had only one room (Table 1).

**Table 1:** Sanitation condition of households in the nomadic population (*n* = 704) of Hadaleala district, Afar region, Northeast Ethiopia

Variables	Frequency	Percent
Drinking water source		
Protected	251	35.7
Unprotected	453	64.3
Drinking water source shared with livestock		
Yes	374	53.1
No	330	46.9
Average per capita water consumption		
≤ 15 l	373	53.0
> 15 l	331	47.0
Water storage container		
Wide mouthed containers	488	69.3
Narrow mouthed containers	216	30.7
Water storage containers were properly covered at the time of the survey		
Yes	26	3.7
No	678	96.3
How to withdrawal water from the storage		
Pouring	684	97.2
Dipping	20	2.8
Home based water treatment		
Yes	34	4.8
No	670	95.2
Common methods of home based treatment		
Chlorination	32	94.12
Sedimentation	2	5.88
Availability of any type of latrine		
Yes	141	20.0
No	563	80.0
Reason for not having latrine		
Temporary settlement	323	57.37
Knowledge gap	155	27.53
Cultural issues	85	15.10
Hand washing after visiting the toilet		
Yes	67	9.5
No	637	90.5
Refuse disposal method		
Controlled disposal	83	11.8
Open field	621	88.2
Types of floor		
Earth floor	594	84.4
Plastered concrete floor	110	15.6
Number of rooms		
One	398	56.5
Two	306	43.5

### Prevalence of childhood diarrheal disease

Mothers were asked about the history of their childhood diarrhea in the past two weeks prior to the data collection period. Accordingly, mothers reported that a total of 184 children had diarrheal disease. Therefore, the two weeks period prevalence of diarrhea among U5C was found to be 26.1% (95% CI: 22.9 - 29.3%). In addition, the occurrence of diarrhea at the time of the survey was also assessed and the report indicated that 81 children had diarrhea at the time of data collection. The point prevalence of diarrhea was therefore 11.5% (95% CI: 9.1, 13.8%).

### Factors associated with childhood diarrheal disease

Types of drinking water sources, condition of water sources, household storage of water, method of withdrawing of water from the container, home based water treatment, covering water storage container, volume of water collected, availability of latrine, presence of faeces in the compound, hand washing after visiting toilet, floor type, number of rooms, and refuse management were variables analyzed by univariable binary logistic regression to choose for multivaraible binary logistic regression analysis on the basis of *p* < 0.2. Types of drinking water sources, condition of water sources, method of withdrawing water from the container, covering water storage container, volume of water collected, availability of latrine, presence of faeces in the compound, hand washing after visiting toilet, floor type and number of rooms had a p – value less than 0.2 by the univariable binary logistic regression analysis. Variables which had a p – value less than 0.2 by the univariable binary logistic regression analysis were then analyzed by multivariable binary logistic regression for controlling the possible effect of confounders. Types of drinking water sources, condition of water sources, volume of water collected, availability of latrine, presence of faeces in the compound, hand washing after visiting toilet and number of rooms were associated with childhood diarrhea by the multivariable binary logistic regression analysis at a *p* < 0.05 (Table 2).

**Table 2:** Sanitation predictors of childhood diarrhea among U5C (n = 704) in Hadaleala district, Afar region, Northeast Ethiopia, May

Environmental variables	Childhood diarrhea		COR with 95% CI	AOR with 95% CI
	Yes	No		
Drinking water source				
Protected	16	235	1	
Unprotected	168	285	8.658(5.040, 14.873)	2.449(1.264, 4.744)^a^
Volume of water collected				
≤ 15 L/C/d	127	246	2.482(1.737, 3.546)	1.535(1.004, 2.346)^a^
> 15 L/C/d	57	274	1	
Water is shared with livestock at the source				
Yes	163	211	11.367(6.985, 18.498)	4.403(2.424, 7.999)^a^
No	21	309	1	
The water storage container is covered at the time of the survey				
Yes	2	24	1	
No	182	496	4.403(1.030, 18.818)	15.681(0.191, 1287.490)
How to withdraw water from the storage				
Pouring	182	502	1	
Dipping	2	18	0.306(0.070, 1.334)	16.464(0.191, 1421.947)
Availability of any type of latrine				
Yes	21	120	1	
No	163	400	2.329(1.415, 3.832)	2.278(1.045, 4.965)^a^
Faeces observed in the compound				
Yes	179	457	4.935(1.953, 12.470)	11.391(2.100, 61.787)^a^
No	5	63	1	
Hand washing after visiting the toilet				
Yes	173	464	1	
No	11	56	0.527(0.270, 1.029)	16.511(3.304, 82.509)^a^
Number of rooms				
Only one room	167	231	12.290(7.248, 20.840)	5.827 (3.208, 10.581)^a^
Two rooms	17	289	1	
Floor type				
Earth floor	168	426	2.317(1.324, 4.053)	2.039 (0.938, 4.434)
Cemented floor	16	94	1	

The result of Hosmer and lemshow test was > 0.05

^a^Statistically significant variables

The current study had illustrated that childhood diarrhea was associated with drinking water sources. The probability of developing diarrheal disease is 2.5 times more likely to be higher among children whose families had collected water from unprotected water sources compared with their counterparts [AOR = 2.449, 95% CI = (1.264, 4.744)]. This community based cross sectional study also depicted that childhood diarrhea was associated with the condition of the sources from where drinking water was collected. For instance, diarrheal disease was highly prevalent if the water sources are shared with livestock [AOR = 4.403, 95% CI = (2.424, 7.999)]. Furthermore, childhood diarrheal disease was statistically associated with the volume of water collected per day. The odds of having diarrhea was 1.5 times more likely to be higher among children whose families collected water below 15 L/C/d compared with children whose families collected water above 15 L/C/d [AOR = 1.535, 95% CI = (1.004, 2.346)].

Human excreta management was associated with childhood diarrheal disease. Children whose families didn’t have any type of latrine were 2.3 times more likely affected by diarrheal disease than children whose families had any type of latrine [AOR = 2.278, 95% CI = (1.045, 4.965)]. Similarly, the odds of having diarrhea was 11.4 times more likely to be higher among children who lived in areas contaminated with human faeces as compared with their counterparts [AOR = 11.391, 95% CI = (2.100, 61.787)]. This study also pointed out childhood diarrhea was associated with the hand washing practice after visiting toilet. Children whose families did not wash their hands after visiting toilet were 16.5 times more likely affected by diarrhea as compared with their counterparts [AOR = 16.511, 95% CI = (3.304, 82.509)].

Diarrheal disease was also associated with housing condition, particularly the number of rooms was associated with childhood diarrhea. Children who lived in houses with only one room had more chance to develop diarrhea than children who lived in houses with two rooms. As depicted by this study, the likelihood of having diarrhea was about 5.8 times more likely to be higher among children who lived in houses with only one room compared with children who lived in houses with two rooms[AOR = 5.827, 95% CI = (3.208, 10.581)].

## Discussion

This community based cross sectional study found that the prevalence of diarrheal disease among the U5C of the nomadic people of Hadaleala district was 26.1% (95% CI: 22.9, 29.3%), which is higher than the national prevalence, 13% [18]. The prevalence of childhood diarrhea reported by this study was higher than the report of other similar studies in Ethiopia, like in eastern Ethiopia (22.5%) [19], Bahir Dar city (21.6%) [20], Mecha district (18%) [21] and Sebeta (9.9%) [22]. On the other, it was lower than the result of studies conducted in Nekemte (28.9%) [23], and Arba Minch district (30.5%) [24]. The variation of results among the studies might be due to the difference in the socio-demographic, environmental and behavioral characteristics of households or mothers, and differences in climatic condition.

The water sources in which the community collected drinking water had a significant effect on the development of childhood diarrheal disease. Children whose families accessed drinking water from unprotected sources had a greater chance to develop diarrhea. This can be explained that unprotected water sources are prone to contamination with human and animal wastes containing a number of diseases causing pathogenic microorganisms which have been associated with increased risks of a range of diarrheal diseases in children. The result of this study was also consistent with the findings of other similar studies [19, 25–30].

The childhood diarrheal disease was associated with the quantity of water collected for daily domestic uses. Those households who had had inadequate service level (below 15 L/C/d) were highly affected by childhood diarrhea.The finding of this study was also in line with the findings of other studies conducted all over the world [31–33]. This might be due to the fact that access to adequate service level has an association with the frequency of hygiene behaviors of the households. It is very difficult to keep the personal hygiene and cleanliness of utensils if there is a shortage of water.

This study had explained that childhood diarrhea was associated with exposure of drinking water sources for livestock. The childhood diarrheal disease was highly prevalent among households whose water source were shared with livestock. Other studies had also reported similar findings with the current study [27, 29]. This could be justified that drinking water sources which are not free from animal contact may contain diseases causing pathogenic microorganisms which come from animal excreta and urine.

Availability of any type of latrine and open defecation were associated with childhood diarrhea. As revealed by this and other similar studies, children lived in areas characterized by open defecation were more affected by diarrheal disease [19–23, 25]. This might be due to the fact that a human excreta contains millions of diseases causing pathogenic microorganisms. Indiscriminately managed human excreta pollutes the living environment and the children may get contaminated by pathogenic microorganisms during crawling, walking and playing. Furthermore, poorly managed human excreta may pollute drinking water sources and different food items and it creates favorable conditions for vector breeding which cause a wide spread of diarrhea.

Households’ hand washing practice after visiting toilet was statistically associated with childhood diarrhea. The importance of hand washing after visiting toilet to minimize the load of microorganisms has been also documented. This could be explained that human hands might be contaminated with microorganisms during defecation and contaminated fingers are the main routes for feco-oral disease transmissions [19, 24, 34–36].

This research showed that childhood diarrheal disease was associated with number of habitable rooms. Childhood diarrhea was more prevalent among households who had only one living room compared with households having two or more living rooms. Similar findings were also reported by earlier studies in developing countries [37, 38]. This might be due to the fact that crowded living rooms may create a chance of close contact among children, which creates a favorable condition for transmission of diseases causing pathogenic microorganisms.

Lastly, even if childhood diarrhea was properly defined using the WHO diarrhea assessment tool, its occurrence was determined based on the report of mothers. It was not confirmed by physicians. Due to this phenomenon, the current study might be affected by social desirability bias. However, strong efforts were made to minimize social desirability bias. Female enumerators who were part of the community were recruited for the purpose of creating strong relationships and links with mothers.

## Conclusions

Childhood diarrheal disease was the common public health problem among nomadic communities in Hadaleala district. Compared with the national and regional prevalence of childhood diarrhea, higher prevalence of diarrhea among U5C was reported in the community. This study had also identified that types of drinking water sources, households whose water sources are shared with livestock, volume of daily water collected, availability of latrine, presence of faeces in the compound, hand washing after visiting toilet and number of rooms were sanitation predictors associated with childhood diarrhea in Hadaleala district, Afar region. Therefore, enabling the community with safe and continuous supply of water and proper disposal of wastes including excreta is necessary with particular emphasis to the rural nomadic communities.

## Abbreviations

AOR: Adjusted Odds Ratio
CI: Confidence Interval
COR: Crude Odds Ratio
Km: Kilometer
Km^2^: Kilometer square
L/C/d: Liter per capita per day
SPSS: Statistical Package for Social Sciences
U5C: Under – five children
UNICEF: United Nations International Children’s Fund
WHO: World Health Organization
